# Caregiver Perspectives on Telehealth Assessment and Other Supports for Infants with Early Developmental Concerns

**DOI:** 10.1007/s10803-024-06483-3

**Published:** 2024-07-27

**Authors:** Daltrey Schmidt, Chloe Urias, Sarah Dufek, Meagan R. Talbott

**Affiliations:** 1 MIND Institute, 2825 50th Street, Sacramento, CA 95817 USA; 2 Department of Psychiatry and Behavioral Sciences, University of California Davis Health, Sacramento, California USA

**Keywords:** Developmental concern, Caregiver, Pre-diagnostic, Infants, Autism

## Abstract

This study examined the experiences of families of children with developmental concerns in the first year of life, before formal diagnostic evaluations are typically conducted. We aimed to understand the impact of participation in a telehealth-based research evaluation in infancy, identify existing community-based supports perceived favorably by caregivers, and identify suggestions for future directions. Participants were recruited from an prior study evaluating a telehealth assessment for infants with early social communication delays. Here, we interviewed caregivers (*n = 19*) who participated in follow-up study in toddlerhood. Transcripts from the semi-structured interviews were transcribed and analyzed using both inductive thematic and content coding approaches. Analysis of these interviews resulted in four core themes describing caregiving during this time: (1) Caregivers felt lonely, overwhelmed, and dismissed by providers, leading to feelings of uncertainty about their child’s development and future; (2) Telehealth assessments were appreciated because external supports are minimal, complex to navigate, and do not address all areas of need; (3) Desire for additional community and connection; and (4) Information is power. Caregivers reported participating in the telehealth assessments helped them to feel reassured, validated and supported. Outside the study, they sought a wide variety of services and resources. The most frequent requests were for parent coaching sessions and family navigation. Caregivers experienced uncertainty and disempowerment during the pre-diagnostic period and sought education and guidance during this time. Findings reflect the importance of centering family priorities when developing early intervention services for infants with elevated likelihood of autism.

The time period between when caregivers first raise developmental concerns and receive formal diagnostic assessment for autism requires families to navigate complex care systems and is associated with elevated parenting stress (DesChamps et al., 2020). Caregivers of autistic children often express concerns about development in the first two years of their child’s life, sometimes as early as 12 months (De Giacomo & Fombonne,1998; Ozonoff et al. 2009). However, there is a significant delay between expression of those concerns to professionals and completion of the evaluation process. Many complex factors contribute to these delays including extensive waitlists at diagnostic clinics, provider hesitancy and diagnostic conservatism (i.e. “wait and see”), and a lack of validated autism-specific screening tools for infants in the first year of life (Kanne & Bishop, 2020; Lemay et al., 2018; Zuckerman et al., 2015). Without an official diagnostic label, many families are unable to access autism-specific services (Gordon-Lipkin et al., 2016). Efforts to improve family support during the pre-diagnostic period^1^ have focused on two areas: improving early identification practices (e.g. screening, triaging, diagnostic models) and development of interventions and supports specifically for the pre-diagnostic infancy period. However, a richer understanding of the perspectives of families navigating this pre-diagnostic period can help to ensure the supports developed adequately address families’ needs. This in turn, will increase the likelihood a novel or adapted existing support will be successfully implemented in practice (Boyd et al., 2022). The goal of the present study is to better understand the lived experiences of families with early developmental concerns in order to learn how to support them during this period of diagnostic uncertainty.

## Current Approaches to Pre-Diagnostic Supports for Infants

A variety of factors contribute to long delays between initial caregiver concern and formal autism diagnosis. Delay between caregivers’ first concerns and formal diagnosis can be multiple years (Crane et al., 2016; Siklos & Kerns, 2007). There are many systemic barriers that may contribute to this delay. In addition to provider hesitancy and long waitlists, these barriers include lack of diagnostic services in suburban and rural areas, financial costs, as well as linguistic and cultural barriers (Blumling et al., 2020; Elder et al., 2016; Zuckerman et al., 2017). Navigating the complex systems involved in obtaining a quality diagnostic evaluation places an enormous burden on families. For example, a mixed-methods analysis identified 66 unique steps involved in the diagnostic process (Broder-Fingert et al., 2019). These barriers are compounded for families of color, who often receive diagnoses later than their white peers (see Fisher et al., 2023 for a systematic review and discussion). Thus, improving system responsiveness to early parental concerns though connection to existing services and development of new supports for the pre-diagnostic period may not only help to improve children’s developmental outcomes but also improve parent and family functioning (Estes et al., 2019). Family navigation approaches may be particularly promising methods for supporting families during the pre-diagnostic period. Navigation involves supporting families in addressing barriers to care (e.g. around transportation, language, etc.), care coordination, and advocacy by peer mentors or other community health professionals. Prior trials have demonstrated the potential for family navigation support to increase families’ participation in diagnostic assessments and reduce time to diagnosis for toddlers in the second year of life (Feinberg et al., 2021; Feinberg, Kuhn, Feinberg et al., 2021a, b).

There are currently no empirically supported interventions specifically for infants with elevated likelihood of autism. In the United States, families with early developmental concerns may be eligible for early intervention services for infants and toddlers with identified delays and/or specific diagnoses through federally funded state grants under Part C of the Individuals with Disabilities Education Act (IDEA; 2004). However, these services are generally low-intensity and not specific to autism and so may not adequately meet the needs of families with specific concerns related to autism. Furthermore, individual states vary in their criteria for determining eligibility, which poses yet another barrier to access (Early Childhood Technical Assistance Center, 2021). In recognition of the need to better support families during this pre-diagnostic window, there has been an increased focus on developing support programs for this period. While some early trials have suggested these pre-diagnostic interventions may help to support infants’ social communication development (e.g.Whitehouse et al., 2021), meta-analyses of the existing evidence base regarding intervention for infants and toddlers with or at elevated likelihood for autism more broadly have not yet reported strong evidence of their efficacy (Hampton & Rodriguez, 2022; McGlade et al., 2023). There is still a need to expand our understanding of families’ unmet needs during this period in order to develop supports that are both efficacious in supporting infant development and acceptable to families and communities. Importantly, it is likely that infants with very early developmental concerns will have a variety of developmental and diagnostic outcomes later in toddlerhood. While some infants with these early concerns will benefit from strategies reducing wait times for evaluations, for many infants, differential diagnosis will not be possible until later in development. Thus, identifying shared areas of concerns and needs of families’ pre-diagnosis has the potential to inform transdiagnostic interventions and supports that may be particularly appropriate for this early developmental period when there are developmental concerns but differential diagnosis is not yet possible.

## The Current Study

The current study was conducted as part of a follow-up project evaluating the acceptability and clinical outcomes of infants who previously participated in a feasibility study of telehealth assessments for infants with early social communication delays and/or specific concern for autism (Talbott et al., 2022). Specifically, the current study was designed to explore families’ perspectives on both the assessment procedures from the feasibility study, as well as their experiences navigating their early developmental concerns more broadly. We interviewed families via telehealth after they had completed a toddler outcome visit in the follow-up study.

Our primary research questions were:

1. What are the shared and unique experiences of families with early developmental concerns?
2. What kinds of supports already provided are most helpful to families?
3. What other supports would they find valuable?

## Methods

### Study Setting

Participants were recruited from a larger longitudinal study evaluating the feasibility and acceptability of a telehealth evaluation for infants with early developmental concerns. Infants were enrolled into this primary study between the ages of 6 to 12 months. Families varied in the extent to which caregivers expressed specific diagnostic concerns (i.e. many but not all had specific concerns related to autism), but all infants met concerns criteria on at least one domain of the Infant-Toddler Checklist (ITC; Wetherby & Prizant, 2002) at initial enrollment. Families were recruited nationally for the initial study through informational postings on University-associated social media sites, child development organization webpages, as well as through dissemination of recruitment flyers to early intervention providers. The total sample of this primary study was 44 across two successive phases. Families enrolled in Phase 1 (*n* = 11) participated in a single telehealth assessment session when their infants were between 6 and 12 months of age. Families enrolled in Phase 2 (*n* = 33) participated in five infant telehealth assessment sessions across a period of approximately 9 months. All families were provided access to online modules for caregivers of toddlers with social communication delays (helpisinyourhands.org). For further details regarding recruitment and sample in the larger study, see Talbott et al., 2022. The current project was part of a follow-up study of these cohorts when infants reached toddlerhood. Families were recontacted and invited to enroll in a follow-up study consisting of two components. First, a 30-month telehealth-based outcome assessment with a clinical psychologist naïve to the infant visits. Outcomes visit measures included (1) the TELE-ASD-PEDS (Corona et al., 2020); (2) the Toddler Autism Symptom Interview (Coulter et al., 2021); and (3) a Clinical Best Estimate rating, based on all available information, of whether toddlers met DSM-5 criteria for Autism, had other clinical concerns (e.g. speech-language delays, ADHD concerns), or were judged to be typically developing. Outcome data on the full primary study sample is forthcoming, but families participating in the exit interview component represented all three of these groups. The second follow-up study component was exit interview with caregivers via telehealth. This exit interview component is the focus of the current study. The research procedures were approved by the IRB at the University of California Davis. Informed consent was obtained from all research participants.

### Participants

Nineteen families enrolled in the follow-up study and completed both the outcome visit and exit interview components and are included in the current study. This included one set of twins, resulting in a final sample of 19 caregivers and 20 infants. Specific demographic information for the subset of families who participated in the exit interviews is presented in Table 1. Although families had varied number of assessment visits in the primary study, we sought to understand the broader experiences of caregivers during this early developmental period. Thus, we included all families who participated in the exit interview component, regardless of whether they had participated in the Phase 1 (*n* = 5) or Phase 2 (*n* = 14) components of the primary study and regardless of toddlers’ specific developmental and diagnostic outcomes.

**Table 1:** Participant demographics

Demographics		
Infant age at initial session (M, SD)		9.10, 2.15
Infant Sex (*n*,* %)*		*N* = 20
Male		7, 35%
Female		13, 65%
Infant Race (*n*, %)		
	Asian	2, 10%
	More than one Race	4, 20%
	White	14, 70%
Infant Ethnicity (*n*, %)		
	Hispanic or Latino	2, 10%
	Not Hispanic or Latino	18, 90%
Maternal Education (*n*, %)		*N* = 19
	High School/GED/Vocational	1, 5.2%
	College Degree	9, 47.4%
	Graduate Degree ^b^	9, 47.4%
Household or Individual Income		
	$9,999 and below	1, 5%
	$25,000 to 49,999	1, 5%
	$50,000 to 74,000	1, 5%
	$75,000 to 99,999	2, 10%
	$100,000 to 124,999	2, 10%
	$125,000 to 149,999	2, 10%
	$150,000 to $174,999	1, 5%
	$175,000 to $199,999	3, 15%
	$200,000 and above	5, 20%
30-Month Outcome Classification (*n*, %)		
	Autism	4, 20%
	Other Clinical Concerns	7, 35%
	Typical Development	9, 45%

Note.^a^ Includes courses towards college and/or associates or 2-year degree ^b^ Includes Masters and Doctorate

### Procedure

Approximately one month after completing the toddler follow-up study, caregivers participated in an exit interview administered over Zoom. A member of the research team trained in qualitative interviewing techniques (e.g., use of open-ended questions, reflective listening practices) engaged in semi-structured conversations following an interview guide outlining nine questions (with accompanying probes if needed) about families’ overall experience with different components of the infant telehealth study, their use of asynchronous online modules, as well their broader experience as caregivers of children with early developmental concerns. The interview guide is presented in Table 2.

**Table 2:** Interview guide including target primary topics and potential follow-up questions

1. I don’t know [child], can you tell me a little bit about them, maybe your favorite activity to do together?
2. Thinking over your entire time in the TEDI study, from the very first visits when [baby] was an infant through your final session with the psychologist, overall, how would you describe your experience in the TEDI study? (RQ1)
3. Thinking about different parts of the study, like the online questionnaires, live telehealth sessions, or written reports you may have received, what was most valuable to you? (why? ) (RQs 1, 2)
4. Do you think the TEDI sessions and reports you received when [name] was a baby helped you access other services you wanted? How? (RQs 2,3)
5. Is there anything you wish this study had done differently or should consider adding? (RQs 1,2,3)
6. Now thinking about the online video modules, what did you think about these materials? (RQ2) a. FQ1: If they did not use, why not? (why wasn’t this useful/appealing? ) Might you have used it if you had access to it earlier? Are there specific topics that might have been more useful to you? Do you think you would have used it if the examples were of younger babies? (RQ 3) b. FQ2: If they did use: how well do you feel the topics addressed your questions? c. FQ3: if they did use: How do you see HIIYH fitting in to this whole process? Would you have used it earlier? Will you continue to use it? What other topics should a resource like this cover? (RQ 3)
7. For families in P1: Thinking back to when [child] was an infant and you first had some questions about their development, would you have liked to have access to online videos and materials with ideas to help support their development? a. If no, why not? b. If yes, what topics would you have liked a resource like that to cover?
8. What would you tell other parents who might become involved in a study like this one? Was anything especially hard that might make it difficult for other families to participate?
9. Is there anything else that I haven’t asked you about that you’d like to tell me?

### Ethics

Informed Consent was obtained from all participants included in the study. All interview videos were stored on a secure HIPAA-compliant and password-protected university drive. De-identified participant code numbers are used in quotations provided below.

### Data Analysis

Analyses began after collection of the first 13 interviews, using a multiple-method approach focused on (a) thematic analysis of caregivers’ experiences and (b) content coding analyses of the frequency of specific supports and strategies they suggested. Initial transcripts were initially autogenerated by closed captioning software during the initial Zoom meeting. Undergraduate research assistants finalized these transcripts by watching video recordings and simultaneously editing the script verbatim to verify dictation accuracy. The cleaned texts were then imported into the program Dedoose for thematic and content analysis (Dedoose, 2021). After familiarizing themselves with the data, the research assistants completed preliminary coding of the interviews by tagging quotes based on commonality, primarily focusing on demographic content and specific feedback about the longitudinal study logistics. This was followed by a discussion with the full author group to discuss overall impressions of families’ experiences and to examine how well the initial code set answered the proposed research questions. This group includes two undergraduate research assistants with a general background in child development and introductory autism training and two established autism researchers with expertise in early identification, infant and toddler development, and intervention and family supports. The group came to an agreement to focus first on Research Question 1 and conduct a full coding pass focused on capturing caregivers’ experiences during this early developmental period. This coding pass utilized a broader, phenomenological lens. Interviews were coded with framing questions such as “*What does this mean or describe about the experience of raising a child with early developmental concerns*?” in mind. Research assistants independently coded the data and organized them into broader themes. The full author group met again to discuss and review the codes and quotes and generate the thematic content and structure. To address RQ’s 2 and 3, research assistants completed a separate coding pass for each transcript, tagging all mentions of specific supports caregivers had either already received or wanted to receive. Responses were tallied and categorized according to type of support. As additional exit interviews were completed, each conversation was transcribed and analyzed for novel content in the same manner detail above. Seventeen interviews were included in thematic analysis, after which thematic saturation was achieved. All nineteen transcripts were analyzed for suggested content supports.

## Results

The thematic analysis focused on caregivers’ experiences during the pre-diagnostic period resulted in four main themes. Each of these is described in turn below, along with illustrative quotes that describe the theme in the words of the participants themselves. Following the presentation of the thematic analyses, we provide a summary of the content coding analyses quantifying participants’ responses regarding existing supports and desired additional supports.

### Thematic Analysis of Caregivers’ Experiences in the Pre-Diagnostic Period

#### Theme 1: Caregivers Felt Lonely, Overwhelmed, and Dismissed by Providers, Leading to Feelings of Uncertainty About Their Child’s Development and Future

Caregivers of children with early developmental concerns were uncertain about the trajectory of their child’s development. Due to the high variability and nonspecific nature of behavior during this infant to toddler period, many caregivers were unsure about which -- if any -- behavioral differences to address. Many families expressed concerns early in infancy. But when they sought feedback from professionals, they did not receive clear answers. Families felt their observations were rapidly dismissed by pediatric providers in particular. Some described feeling ridiculed or shamed for bringing concerns up so early in the child’s life: *“Even her pediatrician would not have even looked at her until she was 3 years old. They won’t even consider it. They think that it’s laughable to have concerns for like a 6 to 9 month old…. It’s been really frustrating to be honest.” [Caregiver #15].* Caregivers were often told to “watch and wait”, but most were not satisfied with this answer. They did not feel their concerns were adequately addressed. Families felt frustrated their experiences were not being taken seriously. Without direction from experts in their community, they felt isolated and helpless: *“Yeah*,* it’s just*,* it’s just hard to talk about. I think it was… frustrating and [a] hard thing to go through and it felt really lonely at times.” [Caregiver #15].* This caused them to feel uncertain about their efficacy as caregivers, as they did not feel fully equipped to understand or support their child’s development. One parent described a feeling of disconnect with her son. This challenged caregivers’ confidence in themselves and was a very frustrating, disempowering experience. *“I don’t know how to put it other than I felt very alone…Yeah*,* I was really I was really lost. I have a daughter*,* she’s 10*,* and the vast differences… I had no clue how to even interact with my son. I was kind of just existing next to him and that’s not how development happens.” [Caregiver #2].*

When reflecting back on this early stage of infancy and toddlerhood, some caregivers wished they had received more education on atypical development and early signs of autism. They felt that this information would have provided some grounding context to their child’s behaviors and mitigated some feelings of uncertainty. Introducing the possibility of autism earlier on could have given them more time to adjust emotionally and smoothed the transition to thinking about their family’s future in a new, different way: *“I can’t remember if they explicitly stated like signs and issues to watch out for in children?…So maybe like one that’s geared specifically*,* to you know*,* females….especially if there are a lot of different elements. Because you have a lot of people who are going to be in denial no matter what.” [Caregiver #16].*

Reconciling the expected, neurotypical trajectory of life with the emerging reality of their child’s unique developmental differences was a complex, difficult process for families. They reported juggling many new logistical changes, including multiple therapy schedules, specialized childcare accommodations, adaptations to their own employment status and significant financial strains. Simultaneously, caregivers were processing their own spectrum of emotions as they began to understand how raising a neurodivergent child would change their lives. Overall, caregivers felt very overwhelmed, both physically and emotionally, as they navigated this adjustment period: *“Yeah*,* it’s just an overwhelming process… on top of that*,* just the emotional stuff too. You know*,* all of a sudden*,* you’re grieving and you’re doing all of those things… you’re working through what a change of what your child’s life you thought was going to be.” [Caregiver #14].*

#### Theme 2: Telehealth Assessments Were Appreciated Because External Supports Are Minimal, Complex to Navigate, and Do Not Address All Areas of Need

During this time, caregivers were actively seeking many forms of support. Of all the supports provided by the present study, families rated the live sessions with examiners (developmental and clinical psychologists) as most helpful. Caregivers liked the ability to connect and converse with a specialized professional trained in early childhood development. This “face-to-face” interaction allowed them to expand on their concerns with nuance and detail that they felt the written form -- like email or questionnaires– did not provide. The next most popular element was the written report received after the live session, which was generated by examiners and included narrative summaries, results of developmental screeners, and individualized developmental goals with suggested activities and strategies for caregivers. Having a succinct summary of their child’s developmental progress from a professional was validating. In some cases, it helped facilitated access to further resources. Caregivers used this report to advocate for additional autism evaluation at their pediatrician’s office or to justify need for speech or occupational therapy at Regional Centers. However, while some families described the long-form questionnaires as educational, the overwhelming majority of felt these forms were too lengthy, detailed, and very time-consuming. They felt this element of the study was not fruitful in terms of providing clarifying answers or conclusions about their child’s development. The study also provided access to online educational video modules for caregivers, “Help is in Your Hands.” Overall, few families independently accessed these online modules. Those that did reported positive experiences with the videos, describing them as very approachable and informative. Caregivers who did not engage with these additional supports indicated the information was redundant with other resources they had obtained, cumbersome to access (not optimized for mobile devices), or required too much time to watch.

When asked the open-ended questions about what other resources they would find helpful, families’ responses were varied. However, nearly all wanted formal recommendations and/or referrals to specialized services that would support them after the conclusion of the study. Given the logistic difficulties and long waitlists for clinics, caregivers wanted guidance towards which accommodations would be most beneficial for their child and worth the significant time investment. Some described wanting a “road map for autism”: *“I think that’s still helpful…even just like suggesting*,* like okay…maybe you should get a speech evaluation…or look at OT for this*,* or maybe put them….in this type of school setting that would be more conducive to help them out*,* you know…like connecting kind of like*,* what to do…parents get really overwhelmed.” [Caregiver #12].*

When asked about what interventions were most helpful, families rated parent coaching-style services very highly. Some discussed their positive experience in the redacted study sessions themselves, while others detailed their experience with other specific parent coaching services. Overall, caregivers enjoyed the experience of interacting with their child and a clinician simultaneously and found the information gained in these types of sessions to be very helpful. They also wanted information on how to navigate their state or local early intervention system at the logistical level. Families with no prior history or knowledge of autism felt especially lost. Although some received support from social workers, they still struggled for a significant period of time with the paperwork, administrative tasks and/or insurance claims required to enroll their children in these necessary services. They felt some information about the length and detail of these processes would have helped them manage their expectations and mitigated stress about establishing prompt care. Families also expressed a desire for more educational materials, specifically information about the criteria and scoring system of measures like the Autism Diagnostic Observation Schedule (ADOS-2; Lord et al., 2012) or Autism Observation Scale for Infants (AOSI; Bryson et al., 2008). They felt this would help them understand their child’s behaviors.

Participants also sought ways to support their family unit as a whole. Not only did they want information on how to parent their neurodivergent child, but they also wanted advice on how to manage interpersonal dynamics between siblings with and without neurodevelopmental differences. While much of the focus was on the child with developmental concerns, caregivers wanted to be equipped to support everyone in their family. Many had a difficult time balancing each sibling’s different needs. They worried about projecting appearances of favoritism onto one child or the other. This was especially difficult when the children were young and did not have the capacity to comprehend the complexity of neurodiversity: *“Well for kiddos that are typical and have siblings who aren’t*,* I think there is like stuff that was brought up to me like with therapists and stuff…they’re gonna feel ignored or feel like it’s not as fair*,* or that we treat the older one differently than we treat the younger one because of his need. Um*,* and like kinda like sibling rivalry.” [Caregiver #13].* When looking for information on how to handle these family dynamics, many caregivers did not feel accurately represented in the resources currently available. They wanted more variety in depictions of neurodiverse families, especially ones where the older sibling is neurodivergent and younger sibling is typically developing. There are many subtle differences in dynamics and sibling social interactions that they felt were not addressed well in the resources they had accessed. They expressed desire for both formal educational materials and informal support groups to help them navigate these challenges: *“…we’re reading all these books about autism and he [speaker’s husband] kept*,* he noticed that a lot of them are….it’s from the perspective written as if it’s always the younger sibling that’s the autistic one*,* and not the older one….And that’s not our situation…And I just think we’re in a different position and I don’t read as much about it.” [Caregiver #4].*

The format in which caregivers consumed or requested for this content was widely varied. Some preferred attending live webinars or Zoom meetings, specifically at times outside regular business hours that are feasible to attend. Others liked more remote options and would rather watch asynchronous videos or read blog posts online. Families also reported regularly consuming parenting content from social media sites like Instagram and Facebook.

#### Theme 3: Desire for Additional Community and Connection

Caregivers desired connection with other neurodivergent families to share resources and build community support systems:*“…I don’t know like a support group*,* or if some kind of*,* you know*,* like occasional webinars or zooms*,* or something about raising a family… with a neurodivergent child…that kind of thing could be interesting… where…information is shared or people can go for support. I think it could be kind of cool.” [Caregiver #4].*

Navigating the diagnostic process and adjusting family dynamics to meet their children’s needs was difficult. People sought emotional support and anecdotal advice from other caregivers to guide their parenting during this period of early development: *“And… it would be nice to -- because we’ve been through the*,* you know*,* the whole process -- to say you know: if you need to talk to someone who’s been through it*,* you know*,* here’s someone who is available to speak with you…” [Caregiver #9].*

Many families also saw participating in research as a way to give back to the neurodivergent community. Some did not have explicit concerns about their child but felt strongly about enrolling in the study as a way to contribute to advancing the understanding of autism and neurodevelopmental disorders. It was very important to them to continue this chain of altruism in order to improve the quality of diagnostic tools and subsequent interventions that kids in the future would receive: *“We wouldn’t have gotten all the information that we got if others didn’t get involved just like we did. The more you get involved*,* the more the community grows*,* the more we understand…And we can be better people to the autistic community.” [Caregiver #2].*

#### Theme 4: Information is Power

Participating in evaluation sessions was a positive experience for caregivers. Being heard and validated by a clinician brought reassurance, regardless of the child’s neurodevelopmental status. Even though formal diagnoses were not made in these early infant-age visits, many people described feeling more informed and at ease, as they began to understand their child’s behaviors better: *“Yeah*,* very informative… And you know I would say things on a fluke*,* and they [clinicians] would say like oh yeah that’s [expected]…I mean jeez you guys give parents*,* guardians*,* caregivers*,* almost like a cape. You know where we feel we’ve got a support team that kind of helps us understand…” [Caregiver #2].*

Some people did not have significant concerns about their child’s development. However, these less explicit concerns did not negate the positive outcomes of the visits. These caregivers reported the feedback provided peace of mind: “*I found it very helpful in the sense that it provided some answers for lingering questions as a parent*,* and it kind of eased my mind in ways*,* that not only where these questions valid*,* but either [child] was right in line with where she needed to be developmentally*,* or maybe it was*,* in fact*,* something that needed to be looked at*,* like speech. So it really provided me peace of mind.” [Caregiver #10].*

Caregivers of children with more serious concerns also described these assessments as validating. Interestingly, feedback given about the child’s developmental differences was described as reassuring, as it legitimized the observations and concerns people had. This was especially impactful for families whose experiences were previously minimized or dismissed by friends, family or pediatric providers: *“He’s very much a tinker. Anyway*,* the questionnaire by far gave me the most comfort. Opened my eyes a little bit… So that was incredible*,* its incredibly… I was telling my husband like “Oh*,* grinding teeth is on here"…And it literally touched on every avenue of his perky little personality from upset to grinding teeth… [interviewer]: …it sounds like those questionnaires were validating– [Parent]: that’s the word I was looking for. Thank you.” [Caregiver #2].*

Overall, caregivers expressed gratitude for being listened to. Although families did not receive diagnostic feedback during the infant visits, they largely reported positive experiences with the assessment sessions. Given the scarcity of screening appointments and systemic lack of access to providers, it is understandable why interactions with clinicians would bring families relief and reassurance: *It was just a depth of information and compassion and almost like we are all gonna be okay. I’m kind of like making sense off of all of it. We’re just*,* I was by the seat of my pants before then. Let’s just say that.” [Caregiver #2]*.

Understanding their child’s development was an empowering experience for caregivers. Many felt better equipped to parent more effectively. This helped them feel more in control of the situation and empowered them to advocate more aggressively for their child in outside settings. Many aspects of life were up-in-the-air, but education provided reassurance and restored peoples’ confidence in themselves. This returned much-needed sense of agency to families’ lives during this very uncertain period of life: *“It gave me confidence…built my confidence. I would say and then I was really feeling like I didn’t have that… so I felt like able to take more control with her… [it] helped me feel like I had*,* I had a resource and I had like an opportunity to take control of it not only to help but also… all over in some ways.” [Caregiver #15].*

### Content Analysis of Frequency of Existing and Desired Supports

#### Existing Supports Endorsed as Helpful

The frequency of each support described by caregivers are presented in Table 3. Overall, caregivers found the live sessions to be the most valuable component of the study. This is consistent with the narratives described above, in which caregivers reported the sessions were both informative and validating. Written reports were also mentioned frequently as being highly valued. They helped families feel validated and provided an additional layer of legitimization at pediatrician’s visits and when requesting evaluations for early intervention services. Few families perceived the questionnaire packets as directly beneficial, though these questionnaires were used in part to generate the provided research report. Finally, approximately one-quarter of caregivers endorsed online video modules as being beneficial. However, very few families had accessed these materials.

**Table 3:** Summary of existing supports endorsed as helpful

Type of Support	Number (Percentage) of Families Endorsing
Live Session	10, 52.6%
Written Report	9, 47.4%
Questionnaire	4, 21.1%
Asynchronous Video Modules	5, 26.3%

### Additional Suggested Supports

The frequency of specific suggested supports is provided in Table 4. In general, responses were variable. However, the most frequent request was for the clinician to provide guidance on how to navigate various administrative logistics of obtaining care: where to sign up for early intervention, school accommodations, cost estimates, insurance coverage, etc. Another suggestion was to provide parent coaching sessions. People appreciated the coaching from the clinician during the assessment visits and wanted to continue learning how to better engage their child during playtime. Many caregivers sought more general information about autism and developmental delays, but in a variety of formats. Some wanted live webinars, while others preferred books, short form videos, or text-based mediums like blogs or emails. A few caregivers suggested even more specific educational materials on early signs of autism and autism diagnostic criteria. They felt being aware of what the clinician is looking for could help them understand their child’s behaviors better. Additionally, caregivers requested resources addressing sibling dynamics between neurotypical and neurodivergent children. They wanted to be equipped to support each individual need, as well as facilitate family cohesiveness. Overall, families had a positive experience in research. Many asked about additional studies open for enrollment, as they expressed desire to continue participating in the research process.

**Table 4:** Summary of suggestions for additional supports

Type of Support	Number (Percentage) of Families Endorsing
Specific recommendations for additional evaluations (OT/PT/speech therapy)	6, 31.5%
Parent coaching services	5, 26.3%
Connection to more studies	3, 15.7%
Info about systemic logistics (How EI works in their state, etc.)	3, 15.7%
Short form informational videos	3, 15.7%
Sibling/family dynamic supports	2, 10.5%
Education about symptoms	2, 10.5%
Educational live webinars	1, 5.2%
Books about autism	1, 5.2%
Appendix of developmental topics	1, 5.2%

## Discussion

This study aimed to explore the experiences of families of infants with developmental concerns during the first year of life. During this very early time period, many caregivers had similar emotional and logistical struggles as previously studied groups of caregivers raising diagnosed autistic children represented in prior literature. However, a major stressor for families in the present study was an overwhelming sense of uncertainty, both about their child’s current behaviors, their identity, and their developmental trajectory. Many caregivers struggled to manage their expectations about their child’s and family’s future without guidance. These gaps in knowledge and care left people feeling isolated and powerless. Importantly, these experiences were shared across families regardless of their infants’ subsequent diagnostic outcomes. This highlights a need for intervention that targets caregivers specifically during this early developmental period, prior to the resolution or consolidation of specific diagnostic profiles. These services have the potential to positively impact caregiver’s confidence and mitigate significant anxiety about the future.

Families did not feel heard by pediatric providers; some even described feeling shamed for raising concerns. This is consistent with previous studies reporting significant delays between expression of first concerns and referral for evaluations even amongst children ultimately receiving a diagnosis of autism (Zuckerman et al., 2015). Although there are currently no empirically supported autism-specific diagnostic tools available for the first year of life, acknowledgment of parent concerns is an important contributor to parents’ satisfaction with the diagnostic process (Braiden et al., 2010). As suggested in Snijder and colleagues (2021), the implementation of family centered, shared-decision making processes should be a high priority for providers. Developing early detection diagnostic tools takes time and expanding access to equitable health care is a complex, systemic issue that will not likely be solved within the next few years. However, taking the time to listen and empathize with families is an immediate action professionals can take and will likely be very effective in improving caregivers’ emotional wellbeing and overall satisfaction with care.

Families in this study sought a variety of supports during this time. They wanted help navigating state and local intervention systems, as well as formal recommendations and referrals to additional therapies their child could benefit from. As noted by Elder and colleagues (2016) there are extensive barriers to accessing care, including financial costs, physical distance, limited availability of educated providers, social stigma, etc. Caregivers in the present study faced many of the same challenges; some reported the process continued to be tedious even after the addition of a case manager or social worker. It is concerning that even the highly resourced families in our study reported challenges understanding and navigating the current care system. Additionally, people requested personalized guidance towards specific supports given their child’s clinical presentation. Many felt overwhelmed and lost in the sea of different referrals and services and wanted a credible source to tell them where to start. These findings suggest that development of family navigation resources specifically for this group of families with very early concerns may be a fruitful first step in terms of developing targeted supports for this group of families. This approach is consistent with existing literature demonstrating the positive impact of family navigation programs for toddlers on reducing the time to diagnosis, as well as feeling more confident and supported (Feinberg et al., 2021a; Levinson et al., 2023).

Participants also requested educational materials about diversity in early developmental milestones, patterns, and trajectories, differential diagnosis of autism versus other neurodevelopmental and developmental disabilities, and management of family dynamics with neurodiverse siblings. The preferred format of these resources varied widely, from asynchronous videos to live webinars to social media posts. Families have busy schedules and consume information in numerous mediums. Therefore, developing materials in diverse formats – written, visual, audio – will help ensure families have access to reliable sources of information. This is a timely challenge, given the rise in social media usage and potential for widespread dissemination of inaccurate or overgeneralized information (Aragon-Guevara et al., 2023).

The most frequently requested additional support was individualized parent coaching sessions. Caregivers appreciated the accessibility of the online format and found the real-time interaction, casual conversation and feedback from the clinician very helpful. They requested additional sessions to learn more specific techniques and collaborative play ideas they could implement in their day-to-day lives. This suggests that current efforts to develop, test, and implement infant interventions via parent coaching are viewed favorably by caregivers and should be prioritized in future research. Some initial studies in this area have demonstrated the potential for these approaches to support infants’ development, though much more work is needed to identify appropriate outcome goals, target populations, and implementation approaches (Dawson et al., 2022; Grzadzinski et al., 2021; McGlade et al., 2023; Rogers et al., 2014; Whitehouse et al., 2021).

Overall, caregivers reported that participating in the study was a positive, fruitful experience. There were several children who developed typically as they aged into toddlerhood, yet caregivers did not express regret raising concerns so early in life or investing time in the study. Families felt heard, validated, and supported. The live telehealth sessions helped them understand their child better. This brought a sense of reassurance, even though a formal diagnosis was not made at that time. These findings reflect a larger sentiment of “information is power.” When people are equipped with knowledge about their child’s development, they become more confident in their efficacy as caregivers. This is an important, empowering experience that can return a much-needed sense of agency back into their lives. These findings show that supports have the potential to not only to have clinical impact, but also benefits for caregiver wellbeing by providing emotional support and mitigating the fear of the unknown.

## Limitations

This study had some important limitations to consider. First, participant responses were retrospectively reported. By the time the exit interviews were conducted, children were between the ages of two and three years and had recently participated in a diagnostic outcome visit as part of the larger study. Some families had previously received diagnostic evaluations outside the study, and for others the toddler visit was their first meeting with a clinical psychologist. Thus, participation in the 30-month study visit may have impacted caregivers’ reflections about the early infancy period, when developmental outcomes were more uncertain. However, the fact that families described positive benefits of early evaluations through the study, regardless of diagnostic outcomes, suggests that this perceived support was not limited to infants with persistent developmental differences. A second limitation of this study is the limited diversity in terms of the socioeconomic and racial, ethnic, and linguistic background of the participants. This limits our understanding of the experiences of families from different backgrounds and the generalizability of our findings. In addition, some participants had an older child with a diagnosis of autism or other developmental delays had prior experience navigating early intervention systems. Importantly, we found that even the highly motivated and resourceful caregivers in this study expressed challenges accessing care. There is the possibility of a response bias in that people with more negative perceptions of their experience in the initial infant study declined to participate in the current follow-up study and interview. Finally, while the interviews were conducted by researchers with no prior relationship with participants, their position as a member of the research team may have biased caregiver responses. However, despite these limitations, this study provides important information about the acceptability and impact of early telehealth assessments as well as unmet needs to address in future research.

## Conclusions

Investigation of families’ lived experiences revealed that the period of early developmental concern was one of great uncertainty, stress and disempowerment. Families reported that telehealth-based assessments provided important support in terms of validating their early concerns and helping empower families to pursue additional desired evaluations and services. Based on their feedback, additional pre-diagnostic supports to prioritize include family navigation, parent coaching, and connection with other families undergoing similar journeys. Focusing on these areas of family priority can help to guide development of interventions and supports that are likely to have high acceptability and likelihood of future implementation.

## Footnotes

1 We use the term pre-diagnostic throughout to reference the time period between parents’ first concerns and formal diagnostic evaluation and/or diagnosis or resolution of concerns. This does not imply that all infants with parent concerns will later be identified as autistic, and in fact, we expect that infants with concerns during this period will have a range of diagnostic and developmental outcomes.
